# A *Lactiplantibacillus plantarum* AL6‐1 Strain Isolated From Air‐Dried Meat Mitigates N‐Dimethylnitrosamine‐Induced Hepatic Injury in Mice

**DOI:** 10.1002/fsn3.70861

**Published:** 2025-09-15

**Authors:** Erke Sun, Xueying Sun, Jin Guo, Lina Sun, Ye Jin, Lihua Zhao, Lin Su

**Affiliations:** ^1^ College of Food Science and Engineering Inner Mongolia Agricultural University Hohhot China; ^2^ Integrative Research Base of Beef and Lamb Processing Technology Inner Mongolia Agricultural University Hohhot China

**Keywords:** alleviation of liver damage, *Lactiplantibacillus plantarum* AL6‐1, N‐dimethylnitrosamine

## Abstract

Excessive intake of N‐dimethylnitrosamine (NDMA) can lead to liver damage and carries a potential carcinogenic risk. This study screened a strain of *Lactiplantibacillus plantarum* ( *L. plantarum* ) AL6‐1 from traditional fermented products in Inner Mongolia. The strain not only has the ability to degrade NDMA efficiently (the degradation rate is 73.62%), but also has better tolerance than other strains, and it has strong adhesion. These functional characteristics were further verified by genome‐wide sequencing. Functional genes related to antioxidant activity, DNA repair, and metabolic regulation were also identified in the genome data, which provided a molecular basis for the protection of the strain against liver injury. Animal experiment results showed that intervention with *L. plantarum* AL6‐1 slowed weight gain in mice, reduced liver index, significantly improved liver tissue structure and reduced the degree of inflammatory cell infiltration, and significantly decreased serum alanine aminotransferase and aspartate aminotransferase levels (*p* < 0.05). Meanwhile, malondialdehyde and NDMA levels in mouse plasma were significantly reduced (*p* < 0.05), while superoxide dismutase and reduced glutathione levels were significantly increased (*p* < 0.05). Additionally, this strain ameliorated liver damage by regulating the expression of hepatic metabolic enzymes Cytochrome P450 2E1, Cytochrome P450 2C37, and Cytochrome P450 1A2. Therefore, the findings of this study provide a theoretical basis for the potential application of *L. plantarum* AL6‐1 in alleviating NDMA‐related liver damage.

## Graphical abstract

Genome analysis identified genes associated with NDMA metabolism and antioxidation. *L. plantarum* AL6‐1 alleviates NDMA‐induced liver injury in mice. *L. plantarum* AL6‐1 regulates liver metabolic enzymes to reduce NDMA toxicity.

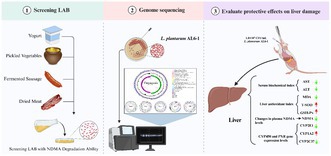

**Abbreviations**
ALDH: aldehyde dehydrogenase
ALT: alanine aminotransferase
AST: aspartate aminotransferase
CYP1A2: cytochrome P450 1A2
CYP2C37: cytochrome P450 2C37
CYP2E1: cytochrome P450 2E1
CYP3A11: cytochrome P450 3A11
CYP450: cytochrome P450
DMEM: Dulbecco's modified Eagle's minimal essential medium
GSH‐Px: glutathione peroxidase
LAB: lactic acid bacteria
MDA: malondialdehyde
NAs: N‐nitrosamines
NDMA: N‐dimethylnitrosamine
PXR: pregnane X receptor
T‐SOD: total superoxide dismutase

## 1 Introduction

Traditional fermented meat products, such as ham, sausages, fermented sausages, and salted fish, are highly favored by consumers for their unique flavors. In cured meat products, nitrates serve as precursors to nitrites, which not only effectively inhibit the growth of pathogens but also prevent lipid oxidation (Shakil et al. 2022). Additionally, nitrites contribute to the development of the unique aroma, color, and flavor of meat products (Majou and Christieans 2018). However, substances such as nitrites and nitrosamines pose potential health risks, particularly liver damage, which has garnered widespread attention (Niklas et al. 2022). Studies have shown that excessive intake of nitrosamines increases the risk of liver cancer (Mitacek et al. 1999).

N‐nitrosamines (NAs) are indirect carcinogens that require metabolic activation by the Cytochrome P450 (CYP450s) enzyme system to exhibit their mutagenic and carcinogenic properties (Seo et al. 2023). NDMA, a type of NA, is metabolically activated by the CYP2E1 isoform of CYP450s in the body, resulting in the hydroxylation of the carbon atom adjacent to the nitrogen atom, forming hydroxynitrosamine. Hydroxynitrosamine is subsequently broken down into aldehydes and hydroxyazo compounds. The latter can cleave into highly electrophilic hydroxy‐diazohydroxide compounds, which bind to DNA to form DNA adducts, causing alkylation damage to DNA bases and ultimately inducing cancer. This process also generates large amounts of intracellular reactive oxygen species (ROS), triggering oxidative stress and leading to liver cell damage (Gao et al. 2017).

Lactic acid bacteria (LAB) are Gram‐positive bacteria widely found in fermented foods and are known for their excellent probiotic functions. They can colonize the gut and improve the intestinal microbiota, thereby alleviating pathological conditions such as gastrointestinal disorders and liver diseases, as well as enhancing the body's immunity (Jeong et al. 2022). Studies have shown that certain strains of LAB can effectively reduce the concentration and genotoxicity of NDMA (Nowak et al. 2014). Rowland and Grasso pointed out that many common gastrointestinal bacteria, particularly those colonizing the stomach and duodenum, can effectively reduce nitrosamine concentrations and inhibit their formation through interactions with secondary amines and nitrites in food. Further research revealed that among the bacteria isolated from the gastrointestinal tract of rats, *Lactobacillus* and *Escherichia coli* strains exhibited stronger nitrosamine degradation capabilities (Rowland and Grasso 1975).

Currently, research on the protective effects of LAB against health damage caused by excessive NDMA intake, particularly liver damage, remains limited. Therefore, screening LAB capable of degrading NDMA and exhibiting excellent probiotic properties, coupled with analyzing their genomic characteristics through whole‐genome sequencing, aims to uncover their potential molecular mechanisms in NDMA metabolism, antioxidation, metabolic regulation, and liver protection. Furthermore, investigating the liver protective effects of LAB in mice fed NDMA‐excessive sausages provides a theoretical basis and practical guidance for developing functional LAB with nitrosamine‐reducing properties.

## 2 Materials and Methods

### 2.1 Materials

A total of 53 strains of LAB were isolated from traditional fermented products in Inner Mongolia (yogurt, pickles, dried meat). Human colon cancer cells (Caco‐2) were provided by the meat science and technology team of Inner Mongolia Agricultural University. Bovine bile salt, NDMA, pepsin, trypsin, penicillin, streptomycin, Sigma Company. Antimicrobial susceptibility paper, Changde Bickman Biotechnology Co. Ltd.; fetal bovine serum, Dulbecco's modified Eagle's minimal essential medium (DMEM), GIBCO, USA. Kits for determining ALT, AST, T‐SOD, GSH‐Px, MDA were obtained from the Nanjing Jiancheng Bioengineering Institute. RNAiso Plus, TaKaRa RR036A reverse transcription kits, and TaKaRa SYBR Premix Ex TaqTM II were provided by Takara Bio (Dalian) Co. Ltd.

### 2.2 Screening LAB With NDMA Degradation Ability

#### 2.2.1 Measurement of NDMA Degradation Ability in Different Strains

The ability of LAB to eliminate NDMA was evaluated by measuring the content of nitrosamines in a co‐culture system using HPLC. Fifty‐three activated strains of LAB were inoculated into MRS medium at a 2% inoculation rate, with a nitrosamine standard added at a concentration of 20 μg/mL to achieve a final NDMA concentration of 1 μg/mL. The cultures were incubated at 28°C or 37°C for 12 h. A control group, without bacterial inoculation, was cultured under the same conditions. The NDMA content in the medium was measured to identify strains capable of effectively reducing nitrosamine levels.

$$\text{NDMA Degradation Rate}=\left(S_{0}-S_{t}/S_{0}\right)\times 100\%$$

In the formula, *S*_*0*_ represents the NDMA content in the supernatant of the control group, while *S*_*t*_ represents the NDMA content in the supernatant of the experimental group.

The detection of NDMA was performed based on Masada's method with slight modifications (Masada et al. 2019). A ZORBAX Eclipse Plus C18 column (4.6 × 250 mm, 5 μm) was used under the following HPLC conditions: mobile phase A was acetonitrile, and mobile phase B was pure water; flow rate: 1 mL/min; injection volume: 10 μL; column temperature: 25°C; detection wavelength: 240 nm. The gradient elution program was as follows: mobile phase A 20% and mobile phase B 80% from 0 to 3 min; mobile phase A 35% and mobile phase B 65% from 3 to 16 min; mobile phase A 90% and mobile phase B 10% from 16 to 25 min; and mobile phase A 20% and mobile phase B 80% at 25 min.

#### 2.2.2 Acid and Bile‐Salt Tolerance Assay for Different Strains

The detection of acid and bile salt resistance in the strains was conducted based on Ramos method with slight modifications (Ramos et al. 2013). Cell suspensions of each strain (2%, v/v) were inoculated into MRS broth adjusted to pH 2.0, 3.0, or 4.0, as well as into MRS broth supplemented with 0.1%, 0.2%, or 0.3% (w/v) oxgall bile salts. Cultures were incubated at 37°C, and samples were collected at 0 and 3 h. Viable counts (CFU/mL) were determined in triplicate by the plate‐count method. Acid‐ and bile‐salt tolerance was inferred from these counts, and survival rates were calculated according to the prescribed formula.

$$\text{Survival Rate}=\left(N_{t}/N_{0}\right)\times 100\%$$

In the formula, *N*_*t*_ represents the viable cell count (CFU/mL) after 3 h of incubation, and *N*_*0*_ denotes the viable cell count (CFU/mL) at 0 h.

#### 2.2.3 Simulated Gastric and Intestinal‐Fluid Tolerance Assay for Different Strains

The detection of gastric and intestinal‐fluid resistance in the strains was conducted based on Zhang's method with slight modifications (Zhang et al. 2016). An aliquot (1.0 mL) of the bacterial suspension of each strain was inoculated into 9.0 mL of simulated gastric juice (pH 2.0) that had been sterilized by passage through a 0.22 μm membrane filter; after thorough mixing, the cultures were incubated anaerobically at 37°C, and viable counts (CFU/mL) were determined at 0 and 3 h. For subsequent intestinal tolerance assessment, 1.0 mL of each suspension was first treated in 9.0 mL of the same simulated gastric juice (pH 2.0) for 3 h, after which 1.0 mL of the treated culture was aseptically transferred to 9.0 mL of simulated intestinal fluid (pH 8.0) that had also been sterilized by 0.22 μm filtration; these mixtures were incubated anaerobically at 37°C, and samples were collected at 0 and 4 h for viable counting (CFU/mL). Survival rates were calculated according to the designated formula. The simulated gastric juice comprised 0.2% (w/v) NaCl and 0.35% (w/v) pepsin, with the pH adjusted to 2.0 using 1.0 mol/L HCl prior to filtration. The simulated intestinal fluid was prepared by mixing pancreatic juice (1.1% NaHCO_3_, 0.2% NaCl, 0.1% trypsin; pH 8.0, sterile‐filtered) with bile (1.2% bovine bile salts; pH 8.0, sterile‐filtered) at a volumetric ratio of 2:1 (v/v).

$$\text{Survival Rate}=\left(N_{t}/N_{0}\right)\times 100\%$$

In the formula, *N*_*t*_ represents the viable cell count (CFU/mL) after 3 or 4 h of incubation, and *N*_*0*_ denotes the viable cell count (CFU/mL) at 0 h.

#### 2.2.4 Adhesion Assay of *L. plantarum* AL6‐1

Caco‐2 were cultured in DMEM medium containing 10% (V/V) heat inactivated fetal bovine serum and 1% (V/V) streptomycin at 37°C and 5% CO_2_. The medium was changed every 2 days. The digested cells were transferred to 12‐well cell culture plate and cultured to monolayer cells. After two generations of activation, *L. plantarum* AL6‐1 was centrifuged, washed twice with sterile PBS, and the bacterial suspension was added to DMEM culture medium without fetal bovine serum and antibiotics. At the same time, the Caco‐2 cell culture plate was washed twice with sterile PBS, and 1 mL of bacterial suspension was added. After incubation for 1 h, the cells were washed gently with sterile PBS for four times to remove non‐adherent bacteria, and 1 mL 1% (V/V) Triton × 100 (room temperature for 10 min and light blowing and mixing) was added to lyse the cells and release adherent bacteria. The adhesion rate was determined by the plate counting method, and the adhesion rate of strain cells was calculated according to the formula.

$$\text{Adhesion Rate}=\left(N_{t}/N_{0}\right)\times 100\%$$

In the formula, *N*_*t*_ denotes the number of adherent colonies (CFU/mL) obtained by plate counting after serial dilution of the lysate; *N*_*0*_ denotes the viable bacterial count (CFU/mL) in the inoculated suspension determined simultaneously.

#### 2.2.5 Antibiotic Susceptibility of *L. plantarum* AL6‐1

Antimicrobial sensitivity was evaluated by the Kirby–Bauer disc‐diffusion method. Briefly, 200 μL of an *L. plantarum* AL6‐1 suspension was evenly spread on MRS agar plates and allowed to air‐dry slightly. Commercial antibiotic discs—norfloxacin, cephaloridine, gentamicin, vancomycin, streptomycin, clindamycin, tetracycline, chloramphenicol, erythromycin, and ampicillin—were then placed equidistantly on the agar surface. After incubation at 37°C for 24 h, the diameters of the inhibition zones were measured to the nearest millimeter (mm).

### 2.3 Extraction and Sequencing of Strain DNA

*L. plantarum* AL6‐1 was streaked onto an agar plate, and a single colony was selected and inoculated into 100 mL of MRS liquid medium. The culture was incubated at 37°C until it reached the logarithmic growth phase. The cells were then harvested by centrifugation at 4°C and 12,000 r/min for 10 min, and the supernatant was discarded. The bacterial pellet was collected and washed with sterile physiological saline. Genomic DNA was extracted using the DM1600 bacterial genomic DNA extraction kit. The extracted DNA was fragmented to approximately 400 bp, and the fragment size distribution was confirmed using agarose gel electrophoresis. A library was constructed using the NEXTFLEX Rapid DNA‐Seq Kit. PacBio sequencing was performed by converting the library into single‐stranded loops, loading it onto the PacBio Sequel IIe sequencer, and sequencing using real‐time detection of light signals during base synthesis.

### 2.4 Quality Control, Assembly and Annotation of Gene Data

Illumina sequencing data were quality‐trimmed using fastp v0.23.0 to obtain high‐quality clean reads. HiFi reads generated by the PacBio Sequel IIe and Illumina data were assembled using Unicycler v0.4.8 and subsequently corrected with Pilon v1.22. Genomic coding sequences (CDS) were predicted using Glimmer and GeneMarkS, while tRNA and rRNA were identified using tRNAscan‐SE and Barrnap, respectively. Functional annotation of the predicted CDS was performed using BLASTP, Diamond, and HMMER against the NR, Swiss‐Prot, Pfam, GO, COG, KEGG, and CAZY databases. The whole‐genome sequencing data in this study have been deposited at the National Center of Biotechnology Information (NCBI). SRA: SRR31513241.

### 2.5 Animal Experiment Design

**FIGURE 1:**
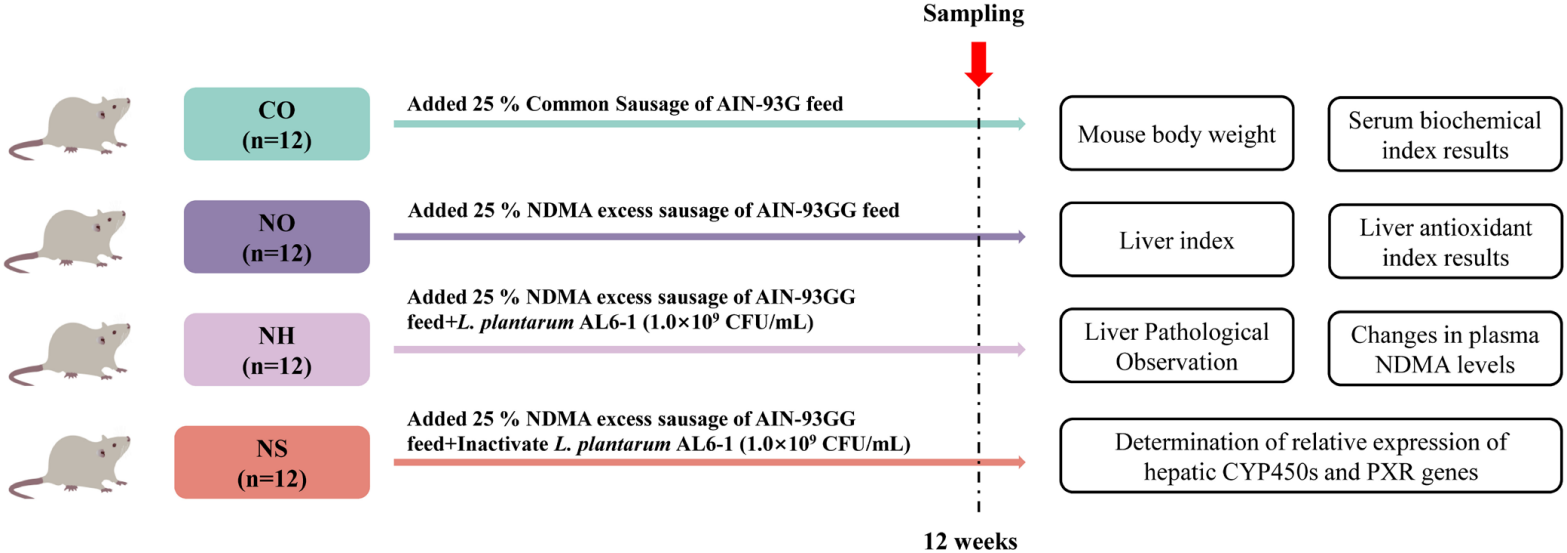
Experiment with animal grouping and feeding.

Male SPF‐grade C57BL/6 mice (8 weeks old, weighing 18–20 g) were purchased from Huhehaote Ruisheng Biotechnology Co. Ltd. (Hohhot, Inner Mongolia, China; license number: SCXK2020‐0005). The mice were housed under controlled conditions: 50% to 60% relative humidity, 20°C to 25°C and a 12‐h light/dark cycle. After a one‐week acclimatization period, the mice were randomly assigned to four groups (12 mice per group): the normal diet group (CO), NDMA diet group (NO), NDMA and *L. plantarum* AL6‐1 group (NH), and NDMA and inactivated *L. plantarum* AL6‐1 group (NS). The CO group was fed a diet containing 25% common sausage added to the AIN‐93G feed. The NO group received a diet containing 25% NDMA‐excess sausage added to the AIN‐93GG feed. The NH group was fed a diet containing 25% NDMA‐excess sausage added to the AIN‐93GG feed supplemented with *L. plantarum* AL6‐1 (1.0 × 10^9^ CFU/mL). The NS group received a similar diet but supplemented with inactivated *L. plantarum* AL6‐1 (1.0 × 10^9^ CFU/mL). The mice were fed their respective diets continuously for 12 weeks. Following a 12 h fasting period, they were euthanized by cervical dislocation, and blood and liver samples were collected for further analysis (as illustrated in Figure 1).

### 2.6 Determination of Liver Index and Histological Examination of Liver Tissue

Mice were euthanized by cervical dislocation, and the liver was quickly dissected, weighed, and rinsed with physiological saline. A portion of the liver tissue was fixed in 4% paraformaldehyde solution for pathological examination. After weighing, the same portion of the liver was dehydrated, embedded in paraffin, sectioned, and stained with hematoxylin and eosin (H&E). The sections were then observed under a light microscope at magnifications of ×200 and ×400. Additionally, the liver index was calculated according to the method described by Gao et al. (2024).

### 2.7 Determination of Serum Biochemical Indicators

Mice were anesthetized by inhalation of excessive ether, and blood was collected via orbital puncture. The blood was centrifuged at 3500 r/min for 10 min, and the serum was separated and stored at −80°C. Serum ALT and AST activities, along with liver oxidative stress indicators, including SOD, GSH‐Px, and MDA, were measured using assay kits following the manufacturer's instructions (Nanjing Jiancheng Bioengineering Institute, Nanjing, Jiangsu, China).

### 2.8 Detection of NDMA Content in Plasma

Mice were anesthetized by inhalation of excessive ether, and blood was collected via orbital puncture. The blood samples were centrifuged at 4°C and 3000 *g* for 10 min to separate the plasma. The NDMA content in the plasma was measured using high‐performance liquid chromatography (HPLC), following the same elution method as that used for screening NDMA‐degrading LAB.

### 2.9 Determination of Relative Expression of Hepatic CYP450s and PXR Genes

Extract total RNA from liver tissue and reverse transcript to obtain cDNA. Gene expression was determined using a LightCycler 96 SW 1.1 real‐time quantitative PCR instrument. The primer sequences of the relevant genes are shown in Table 1.

**TABLE 1:** Quantitative real‐time PCR primers.

Gene	Sequence (5′–3′)
β‐Actin	F: TGTTACCAACTGGGACGACA
	R: GGGGTGTTGAAGGTCTCAAA
CYP2E1	F: CCTGCTGCCCATCATTATCC
	R: GCTCTTACCCACTGAGCCATCT
CYP1A2	F: TGGAGCTGGCTTTGACACAG
	R: CGTTAGGCCATGTCACAAGTAGC
CYP2C37	E: CTGCATGACAGCACGGAGTT
	R: GTGGCCAGGGTCAAATTTCTC
CYP3A11	E: ACAACAAGCAGGGATGGAC
	R: GGTAGAGGAGCACCAAGCTG
PXR	F: GTTCAAGGGCGTCATCAACT
	R: TCGTGTTGAACCTCAGGATG

### 2.10 Statistical Analysis

All experiments were performed in triplicate and analyzed using GraphPad Prism software (version 8.3.0). Experimental results were expressed as mean ± standard deviation ($\bar{X}$ ± SD). Statistical analysis was conducted using one‐way analysis of variance (ANOVA), with significance set at *p* < 0.05 for all tests.

## 3 Result

### 3.1 Screening NDMA‐Reducing LAB and Evaluation of Their Probiotic Properties

#### 3.1.1 Effects of Different Strains on NDMA Content

A total of 53 LAB strains were co‐cultured with NDMA in MRS medium, and the nitrosamine content was determined using HPLC. As shown in Table S1, the NDMA degradation rates of these strains ranged from 5.55% to 73.62%. The ability to degrade NDMA varied among the strains, consistent with the findings of Kim, which may be attributed to individual differences among LAB (Kim et al. 2017). Four strains with NDMA degradation rates exceeding 60% were identified: *L. plantarum* AL6‐1 (73.62%), *Pediococcus pentosaceus* 37X‐11 (68.93%), *Lactobacillus curvatus* SL‐1 (66.51%), and *P. pentosaceus* 37X‐13 (61.95%). These strains showed significantly higher degradation rates compared to the others (*p* < 0.05). Consequently, these four strains with strong NDMA degradation capabilities were selected for further research.

#### 3.1.2 Tolerance of Strains

As shown in Table 2, *L. plantarum* AL6‐1 exhibited high survival under various stress conditions: a survival rate of 58.21% in an acidic environment at pH 2.0, 61.32% in the presence of 0.3% bile salts, 62.22% after 3 h of exposure to simulated gastric fluid, and 59.82% after 4 h of exposure to simulated intestinal fluid. All these values were significantly higher than those of other strains (*p* < 0.05), indicating that this strain is capable of surviving gastrointestinal conditions and reaching the small intestine to exert its effects. The Caco‐2 cell line, derived from human colon adenocarcinoma, is widely used as an in vitro model of the human intestinal epithelium. *L. plantarum* AL6‐1 adhered to Caco‐2 monolayers at a rate of 10.18% ± 0.23%, comparable to the value reported by Behbahani et al. (2019), indicating that the strain exhibits good epithelial adhesion and intestinal colonization potential. Antibiotic‐susceptibility testing (Table 3) revealed that *L. plantarum* AL6‐1 was sensitive to clindamycin, chloramphenicol, erythromycin, tetracycline, and ampicillin, but exhibited intrinsic resistance to norfloxacin, gentamicin, streptomycin, and vancomycin. The latter pattern concurs with the chromosomally encoded, vertically transmitted resistance to streptomycin and vancomycin commonly observed in LAB (Gueimonde et al. 2013). Collectively, these results demonstrate that *L. plantarum* AL6‐1 possesses robust gastrointestinal tolerance, appreciable epithelial adhesion, and an acceptable safety profile, making it a promising functional probiotic candidate.

**TABLE 2:** LAB tolerance survival rate (%).

Strain number		AL6‐1	SL‐1	37X‐11	37X‐13
LAB acid tolerance	pH 2.0	58.21 ± 0.06^Ac^	16.46 ± 0.04^Bc^	10.38 ± 0.02^Cc^	16.44 ± 0.04^Bc^
	pH 3.0	64.32 ± 0.04^Ab^	32.63 ± 0.13^Cb^	28.31 ± 0.12^Db^	35.12 ± 0.07^Bb^
	pH 4.0	73.56 ± 0.14^Aa^	57.92 ± 0.04^Ba^	53.95 ± 0.06^Ca^	57.90 ± 0.04^Ba^
LAB bile tolerance	0.1% Bile salt concentration	89.23 ± 0.17^Aa^	72.11 ± 0.09^Ba^	68.77 ± 0.13^Da^	69.38 ± 0.05^Ca^
	0.2% Bile salt concentration	72.46 ± 0.09^Ab^	57.01 ± 0.12^Bb^	52.36 ± 0.14^Db^	56.30 ± 0.16^Cb^
	0.3% Bile salt concentration	61.32 ± 0.04^Ac^	32.95 ± 0.12^Bc^	24.10 ± 0.06^Cc^	33.03 ± 0.08^Bc^
LAB simulation of artificial gastric acid tolerance	Simulate artificial gastric juice	62.22 ± 0.14^A^	26.33 ± 0.22^B^	23.31 ± 0.09^D^	25.34 ± 0.14^C^
LAB simulation of artificial intestinal fluid tolerance	Simulated artificial intestinal fluid	59.82 ± 0.23^A^	28.68 ± 0.08^B^	21.34 ± 0.11^C^	28.65 ± 0.10^B^

*Note:* Absence of identical uppercase letters in the same row indicates significant differences (*p* < 0.05) among different groups within the same experimental period. Absence of identical lowercase letters in the same column indicates significant differences (*p* < 0.05) within the same group across different periods.

**TABLE 3:** Criteria for determining the types of antibiotics, drug sensitivity paper content, and drug sensitivity circle diameter.

Antibiotic name	Drug quality (μg)	Criteria for determining the diameter of drug susceptibility circle/mm			Sensitivity
		*R*	*I*	*S*	
Norfloxacin	10	≤ 12	13–16	≥ 17	*R*
Cefradine	30	≤ 14	15–17	≥ 18	*I*
Gentamicin	10	≤ 12	13–14	≥ 15	*R*
Streptomycin	10	≤ 11	12–14	≥ 15	*R*
Vancomycin	10	—	—	≥ 15	*R*
Clindamycin	2	≤ 14	15–19	≥ 20	*S*
Tetracycline	30	≤ 11	12–14	≥ 15	*S*
Erythromycin	15	≤ 13	14–22	≥ 23	*S*
Ampicillin	10	≤ 13	14–16	≥ 17	*S*
Chloramphenicol	30	≤ 12	13–17	≥ 18	*S*

*Note:* *R* (Resistance) represents drug resistance; *I* (Intermediate) represents moderate sensitivity; *S* (Sensitive) represents sensitivity; “—” represents the diameter of the drug sensitive circle; the diameter of the drug sensitive tablet is about 6 mm.

### 3.2 Genome Analysis of *L. plantarum* AL6‐1

#### 3.2.1 Genome Overview

**FIGURE 2:**
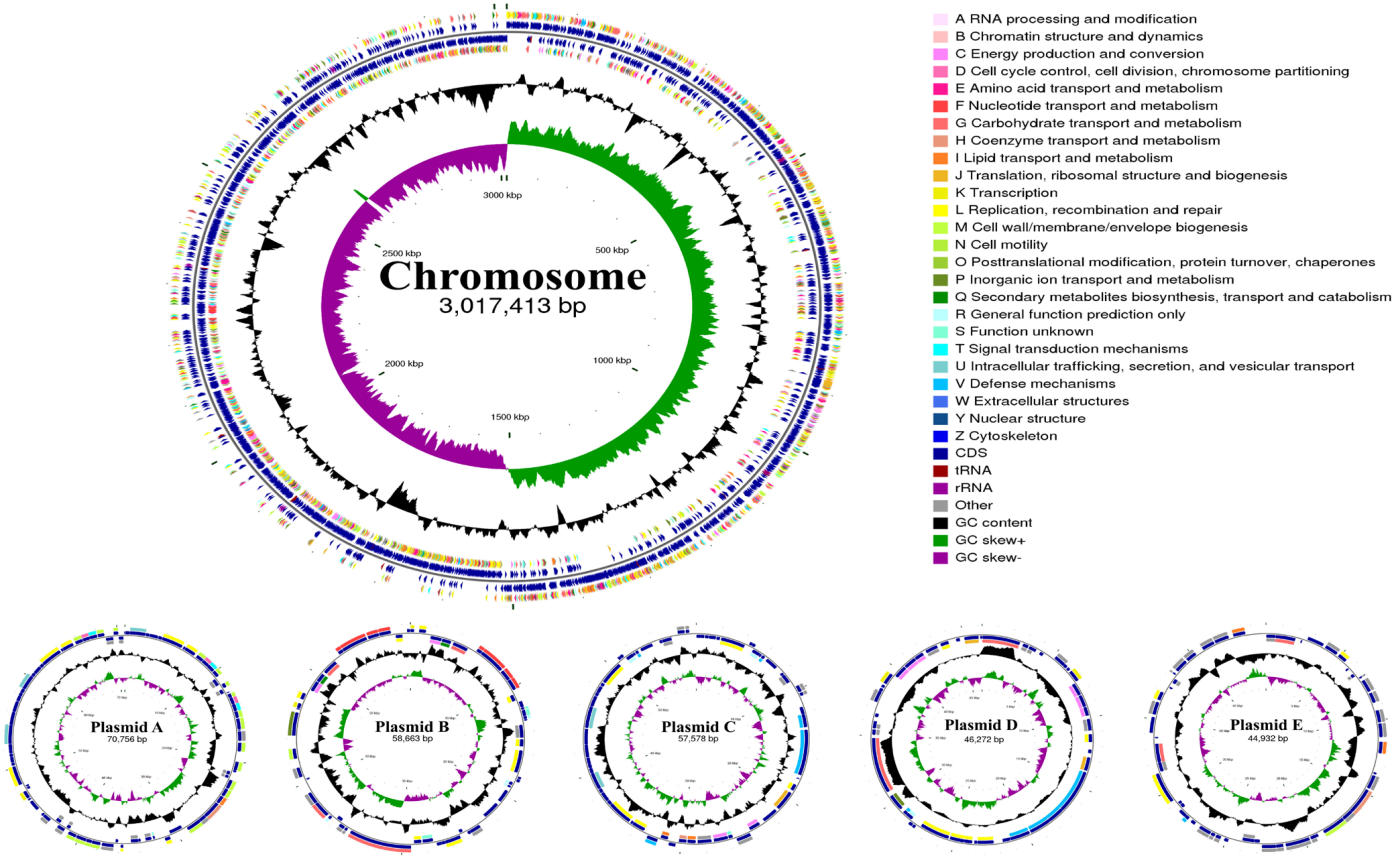
Genomic circle map of *L. plantarum* AL6‐1 strain. The first and fourth rings represent the CDS on the positive and negative strands. The second and third rings represent tRNA and rRNA genes on the positive and negative strands. The fifth ring shows the GC content. The sixth ring represents the GC‐skew value. The innermost ring indicates genome length markers.

**FIGURE 3:**
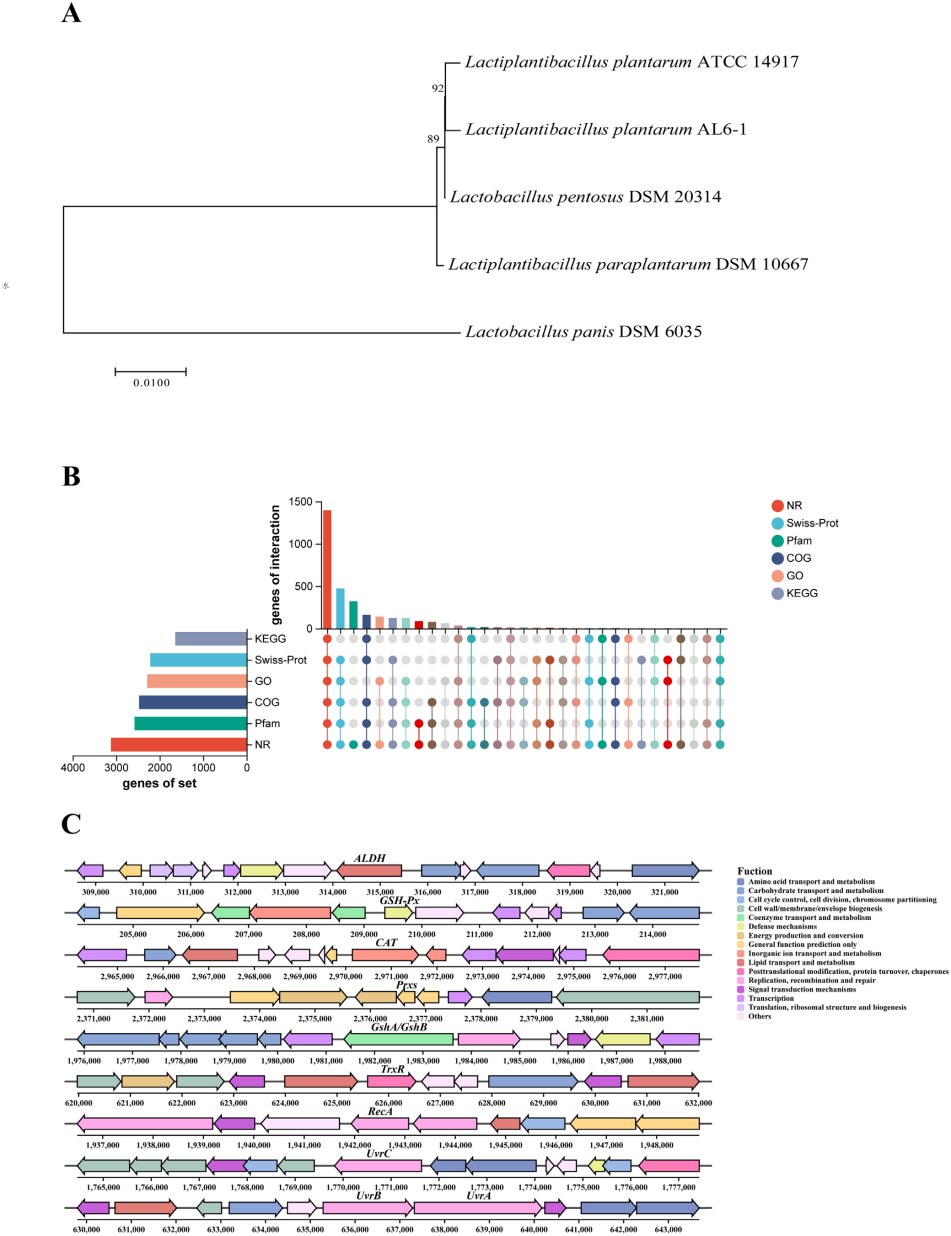
Genetic relationship, gene function classification, and functional gene cluster of *L. plantarum* AL6‐1 (A) Evolution tree of *L. plantarum* AL6‐1 strain. (B) Functional annotation of the coding genes of *L. plantarum* AL6‐1 (Functional annotation of *L. plantarum* AL6‐1 coding genes: The left bar chart shows the total genes annotated by each database, while the matrix highlights unique (single dots) and shared (connected dots) annotations. The top bar chart indicates the number of unique or shared genes for each combination.). (C) Gene clusters related to NDMA metabolism and their annotations in *L. plantarum* AL6‐1.

To gain deeper insights into the genetic mechanisms of *L. plantarum* AL6‐1 in NDMA metabolism, antioxidation, metabolic regulation, and liver protection, whole‐genome sequencing was conducted using a combination of Illumina second‐generation and PacBio third‐generation sequencing technologies. The results revealed that the genome of *L. plantarum* AL6‐1 is 3,295,614 bp in length, consisting of a single circular chromosome and five plasmids, with an average GC content of 44.41% (Figure 2). Phylogenetic analysis showed that the closest evolutionary relative of *L. plantarum* AL6‐1 is *Lactiplantibacillus plantarum* ATCC 14917 (Figure 3A). A total of 3123 coding sequences (CDS) were identified, spanning 2,762,802 bp and accounting for 83.83% of the total genome length. Additionally, among the non‐coding RNAs, 68 tRNA genes, 16 rRNA genes, and 48 sRNA genes were identified (Table S2).

#### 3.2.2 Functional Annotation

Whole‐genome analysis of *L. plantarum* AL6‐1 identified 3123 CDS, which were annotated with various functions using multiple databases (Figure 3B), including KEGG (1641), GO (2284), COG (2474), Pfam (2577), Swiss‐Prot (2217), and NR (3120).

These genes are associated with diverse functions, such as antioxidative stress, energy metabolism, cell repair, and other critical processes. The genome contains 41 genes related to amino acid metabolism, which play key roles in regulating oxidative stress, repairing damaged hepatocytes, and maintaining liver function (Moura et al. 2017). Additionally, 82 genes associated with carbohydrate metabolism support energy production through pathways like glycolysis and the tricarboxylic acid (TCA) cycle, aiding the liver's detoxification and repair functions (Gonzalez and Betts 2019). Furthermore, the genome encodes 5 genes involved in coenzyme metabolism, including those for coenzyme A, NADH (reduced nicotinamide adenine dinucleotide), and NADPH (reduced nicotinamide adenine dinucleotide phosphate). These coenzymes play significant roles in fatty acid metabolism and antioxidative reactions, effectively reducing oxidative stress in hepatocytes (Dong et al. 2023). The synergistic effects of these metabolic pathways provide a molecular basis for *L. plantarum* AL6‐1's ability to alleviate liver injury.

#### 3.2.3 Environmental Adaptability Related Genes

*L. plantarum* AL6‐1 demonstrated strong acid and bile salt tolerance. Genome annotation revealed that this strain encodes multiple molecular chaperone proteins, including GroES, GroEL, GrpE, Hsp33, DnaK, DnaJ, FtsH, and the Pta‐AckA metabolic pathway, which are associated with protein repair and degradation (Table S3). These genes play a critical role in enabling resistance to acid and bile stress (Liu et al. 2023). Additionally, the genome of *L. plantarum* AL6‐1 encodes seven F0F1‐ATPase‐related proteins, five mucin‐binding domains (MucBP: PF06458, PF12799, PF19258, PF03382, PF13306), and two collagen‐binding domains (PF01391, PF05737). These proteins are instrumental in maintaining intracellular pH homeostasis and enhancing the strain's adhesion to the intestinal mucosa, contributing to its adaptability and stability in the gut (Boucard et al. 2022).

#### 3.2.4 Genes Related to NDMA Metabolic Pathways

Whole‐genome analysis of *L. plantarum* AL6‐1 identified key functional genes involved in NDMA metabolism, antioxidation, and DNA repair (Figure 3C). Aldehyde dehydrogenase (ALDH, gene 0290) indirectly contributes to NDMA metabolism by converting toxic intermediates, such as formaldehyde, into harmless substances that can be excreted from the body (Dalle‐Donne et al. 2006). DNA repair genes, including recombinase A (gene 1879) and UV resistance proteins A, B, and C (genes 0598, 0597, and 1697), address DNA damage caused by oxidative stress and NDMA, thereby maintaining genomic stability (Machius et al. 1999; Pan et al. 2024). Additionally, antioxidant‐related genes, such as glutathione peroxidase (gene 0189), catalase (gene 2859), and peroxiredoxin (gene 2294), protect cells by scavenging free radicals and mitigating oxidative stress generated during NDMA metabolism (Ali et al. 2020). Collectively, these genes provide a molecular basis for the ability of *L. plantarum* AL6‐1 to alleviate NDMA‐induced liver damage.

### 3.3 Effect of *L. plantarum* AL6‐1 on Liver Injury in Mice Caused by NDMA

#### 3.3.1 Growth and Weight Change of Experimental Mice

**FIGURE 4:**
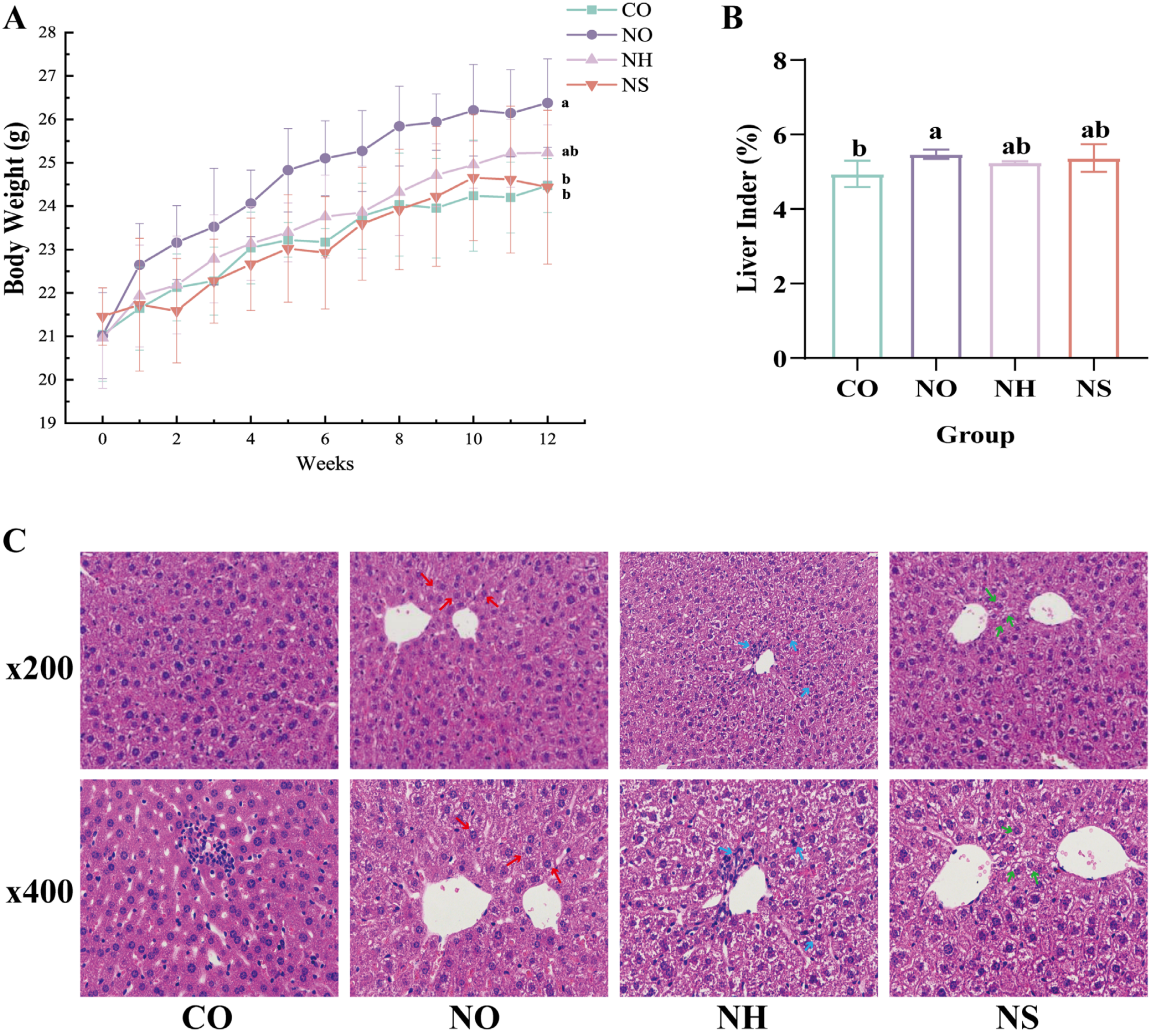
Effects of *L. plantarum* AL6‐1 on mouse body weight, liver index, and histopathological structure. (A) Body weight of mice in each group during the experiment. (B) The liver index of each group during the experiment (%). (C) Liver pathological observation. Different lowercase letters indicate significant differences between different groups (*p* < 0.05). Red arrows indicate the NO group, blue arrows the NH group, and green arrows the NS group, with the CO group left unlabeled. The arrows denote cytoplasmic shrinkage, chromatin condensation, and inflammatory cell infiltration. CO: Added 25% Common sausage of AIN‐93G feed; NO: Added 25% NDMA excess sausage of AIN‐93GG feed; NH: Added 25% NDMA excess sausage of AIN‐93GG feed + *L. plantarum* AL6‐1 (1.0 × 10^9^ CFU/mL); NS: Added 25% NDMA excess sausage of AIN‐93GG feed + inactivate *L. plantarum* AL6‐1 (1.0 × 10^9^ CFU/mL).

The general health status of animals provides a visual indication of their nutritional condition during growth. In this experiment, mice in all groups exhibited smooth fur, normal appetite, and good overall growth. The model group was established by feeding mice with NDMA‐excess sausage feed for 12 weeks. The weekly weight gain of mice in each experimental group is shown in Figure 4A. The results revealed that during the first 4 weeks, the body weight of mice in all experimental groups increased rapidly. After the fourth week, the rate of weight gain slowed in all groups, possibly due to the higher appetite and faster growth rate observed in younger mice. In contrast, during later developmental stages, weight gain naturally slows as the mice approach a stable growth phase (Hao et al. 2022).

#### 3.3.2 Liver Index of Experimental Mice

The liver index is an indicator of liver function impairment, with an increased liver index suggesting a higher degree of liver damage (Liu et al. 2019). The changes in the liver index for each group of mice are presented in Figure 4B. The results show that the liver index of all experimental groups was higher than that of the CO group, particularly in the NO group. This is likely due to oxidative stress‐induced weight loss and liver swelling, or lipid accumulation and lesions in the liver caused by feeding the mice NDMA‐excess sausage feed. The liver index of the NO group was significantly higher than that of the CO group (*p* < 0.05), while no significant difference was observed between the CO and NH groups (*p* > 0.05). These findings suggest that gavage administration of *L. plantarum* AL6‐1 can mitigate liver damage to some extent.

#### 3.3.3 Liver Pathological Observation

HE staining is commonly used to detect and evaluate organ damage. The pathological liver sections of mice from each group, observed at ×200 and ×400 magnifications, are shown in Figure 4C. The CO group exhibited intact hepatocyte structures with centrally located nuclei and no significant atypical changes. In contrast, the NO and NS groups showed severe liver damage, including disrupted tissue structures, disorganized hepatocytes, uneven cytoplasmic staining, nuclear size variability, diffuse lymphocytic infiltration, and marked inflammation. These pathological changes are the primary reasons for the increased liver index (Lu et al. 2021). Compared with the NO and NS groups, the NH group demonstrated significant improvement in vacuolar degeneration and inflammatory cell infiltration. These findings suggest that the whole cell lysate of *L. plantarum* AL6‐1 can mitigate the progression of liver lesions induced by nitrosamines in mice.

#### 3.3.4 Serum Biochemical Index Results

**FIGURE 5:**
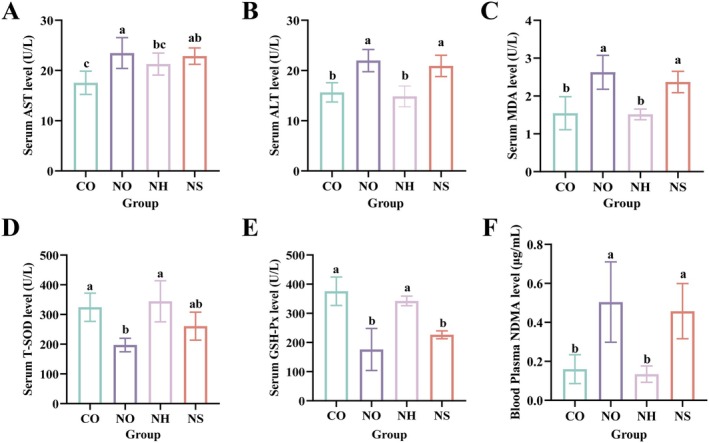
Effects of *L. plantarum* AL6‐1 on serum biochemical indexes and plasma NDMA in mice: (A) serum AST level; (B) serum ALT level; (C) serum MDA level; (D) serum T‐SOD level; (E) serum GSH‐Px level; (F) blood plasma NDMA level. Different lowercase letters indicate significant differences between different groups (*p* < 0.05). CO: added 25% common sausage of AIN‐93G feed; NO: added 25% NDMA excess sausage of AIN‐93GG feed; NH: added 25% NDMA excess sausage of AIN‐93GG feed + *L. plantarum* AL6‐1 (1.0 × 10^9^ CFU/mL); NS: added 25% NDMA excess sausage of AIN‐93GG feed + inactivated *L. plantarum* AL6‐1 (1.0 × 10^9^ CFU/mL).

Serum AST and ALT levels are critical clinical indicators of liver function. Liver damage leads to the release of AST and ALT from liver tissues into the bloodstream, resulting in elevated serum levels (Kou et al. 2020). The effects of *L. plantarum* AL6‐1 on serum AST and ALT levels are presented in Figure 5A,B. The AST and ALT levels in the NO group were significantly higher than those in the CO group at various experimental stages (*p* < 0.05), indicating that excessive NDMA intake causes liver damage in mice and confirming the successful establishment of the liver injury model. In contrast, the AST and ALT levels in the NH group were significantly lower than those in the NO group (*p* < 0.05). These findings suggest that *L. plantarum* AL6‐1 significantly alleviates liver damage induced by excessive nitrosamine sausage consumption.

#### 3.3.5 Liver Antioxidant Index Results

NDMA can induce cancer through metabolic activation by CYP450 enzymes in vivo, a process that generates large amounts of reactive oxygen species (ROS), leading to oxidative stress and liver cell damage (Santos et al. 2014). To evaluate oxidative stress, key markers such as MDA, T‐SOD, and GSH‐Px were measured in mouse serum, as shown in Figure 5C–E. The MDA levels in the NO group were significantly higher than those in the CO group, while T‐SOD and GSH‐Px levels in the NO group were significantly lower than those in the CO group (*p* < 0.05). This indicates that long‐term feeding of NDMA‐excess sausage induces hepatic lipid peroxidation and disrupts the oxidative/antioxidative balance, leading to chronic liver injury caused by oxidative stress. In contrast, the NH group showed significantly lower MDA levels and higher GSH‐Px levels compared to the NO and NS groups (*p* < 0.05), with no significant difference in GSH‐Px levels compared to the CO group (*p* > 0.05). These results suggest that intervention with live *L. plantarum* AL6‐1 effectively inhibits hepatic lipid peroxidation, reduces lipid oxidation product formation, and increases hepatic antioxidant enzyme levels. This enhances free radical scavenging capacity and alleviates liver injury (Ma, Lin, et al. 2024; Ma, Wu, et al. 2024).

#### 3.3.6 Changes in Plasma NDMA Levels

After feeding mice with NDMA‐excess sausage, NDMA is primarily absorbed in the upper small intestine, with some absorption occurring in the stomach. It then enters the liver through the bloodstream for further metabolism (Owiti et al. 2022). The NDMA concentrations in mouse plasma are shown in Figure 5F. The plasma NDMA levels in the NO and NS groups were significantly higher than those in the other groups, whereas the NH group showed significantly lower levels compared to the NO group (*p* < 0.05). These findings indicate that feeding mice NDMA‐excess sausage significantly increases NDMA concentrations in plasma. However, gavage administration of *L. plantarum* AL6‐1 effectively reduces plasma NDMA levels, likely by promoting NDMA metabolism and excretion.

#### 3.3.7 Role of *L. plantarum* AL6‐1 on CYP450 and PXR

**FIGURE 6:**
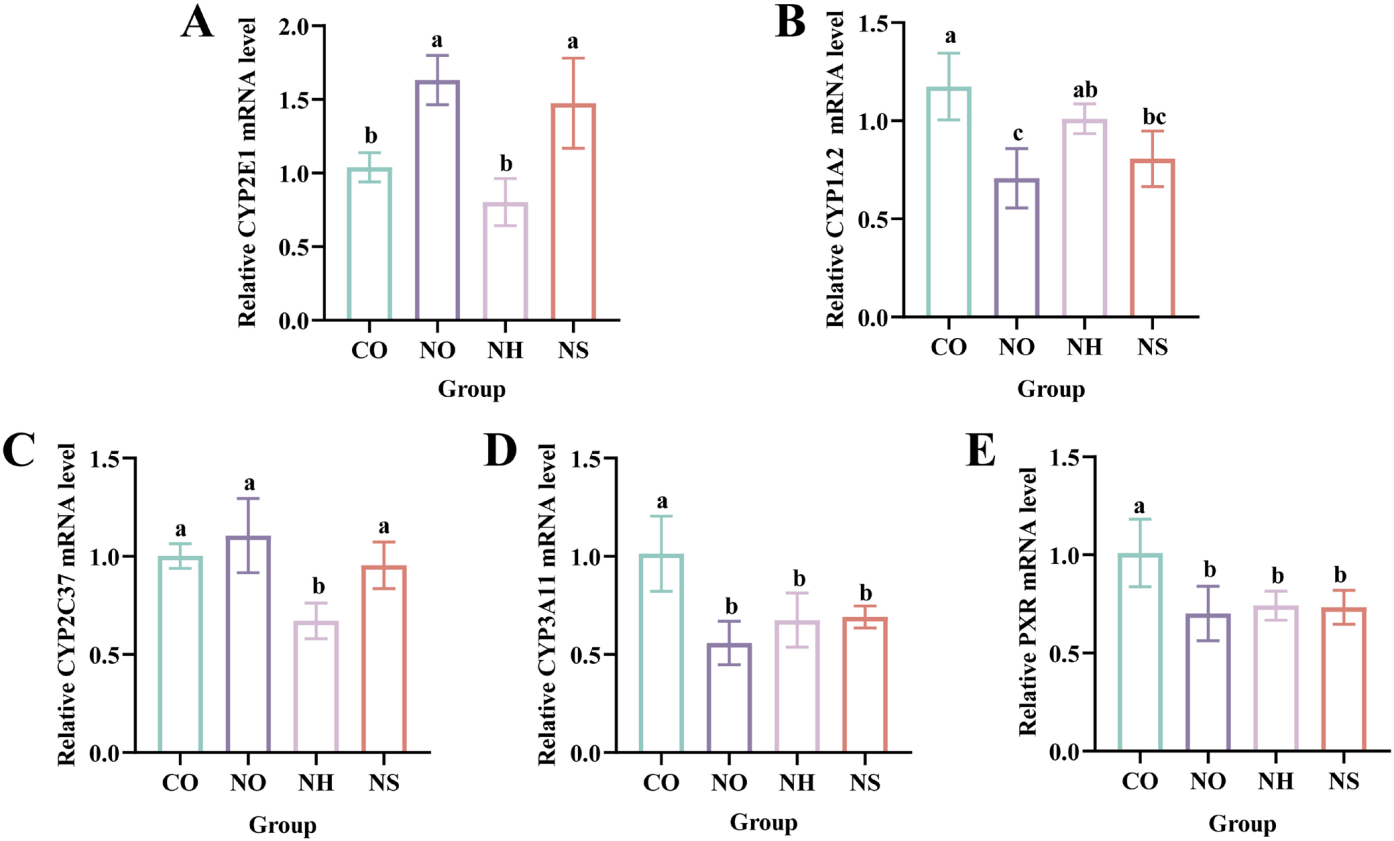
Role of *L. plantarum* AL6‐1 on CYP450 and PXR (A–E) CYP2E1, CYP1A2, CYP2C37, CYP3A11, PXR mRNA level in liver. Different lowercase letters indicate significant differences between different groups (*p* < 0.05). CO: added 25% common sausage of AIN‐93G feed; NO: added 25% NDMA excess sausage of AIN‐93GG feed; NH: added 25% NDMA excess sausage of AIN‐93GG feed + *L. plantarum* AL6‐1 (1.0 × 10^9^ CFU/mL); NS: added 25% NDMA excess sausage of AIN‐93GG feed + inactivated *L. plantarum* AL6‐1 (1.0 × 10^9^ CFU/mL).

As shown in Figure 6A–C, the mRNA expression levels of CYP2E1 in the NH and CO groups were significantly lower than those in the NO and NS groups (*p* < 0.05). The expression of the CYP1A2 gene in the NH group was significantly higher than in the NO group (*p* < 0.05), whereas the expression of CYP2C37 in the NH group was significantly lower than in the other three groups (*p* < 0.05). These findings suggest that *L. plantarum* AL6‐1 regulates the mRNA expression of CYP2E1, CYP1A2, and CYP2C37, thereby protecting mice from liver injury induced by excessive NDMA. This aligns with Zhao's findings, where *L. plantarum* C88 was shown to mitigate chronic alcoholic liver injury by downregulating CYP2E1 gene expression (Zhao et al. 2017). As shown in Figure 6D,E, the gene expression levels of CYP3A11 and PXR did not differ significantly among the NO, NH, and NS groups (*p* > 0.05), but all were significantly lower than those in the CO group (*p* < 0.05). These results indicate that feeding sausages containing excessive NDMA reduces CYP3A11 and PXR gene expression, and intervention with *L. plantarum* AL6‐1 does not affect the expression of these genes.

## 4 Discussion

NDMA is a compound with significant carcinogenic potential, and excessive intake poses serious risks to liver health. Research has shown that NDMA is primarily metabolized in the liver, where its toxic metabolites can damage liver cells, trigger inflammatory responses, and impair liver function. Nitrification reactions commonly occurring in processed meat products—such as interactions between nitrites, nitrogen oxides, and biogenic amines—are key pathways for NDMA formation (Xiao et al. 2018). Existing studies highlight the beneficial effects of LAB in alleviating liver diseases. These bacteria not only regulate the host's microecological balance but also colonize the gastrointestinal tract, providing sustained probiotic benefits (Jeong et al. 2022). Consequently, developing a microecological preparation that mitigates NDMA‐induced liver damage while offering overall health benefits has become a major research focus. This study, a strain of LAB with high NDMA degradation ability and potential probiotic properties, was screened, and its key functional genes were analyzed by whole genome sequencing to further explore its role in alleviating NDMA‐induced liver injury in mice.

A total of 53 LAB strains isolated from traditional fermented products in Inner Mongolia were co‐cultured with NDMA in MRS medium. The nitrosamine content in the medium was measured using HPLC, and the NDMA degradation rate of each strain was calculated. The results identified four strains of LAB with degradation rates exceeding 60%. According to Kim, *Leuconostoc carnosum* , *Leuconostoc mesenteroides* , *L. plantarum* , and *Lactobacillus sakei* exhibit significant NDMA degradation capabilities, effectively reducing NDMA content in both culture media and fermented foods. These effects are likely due to their ability to directly degrade NDMA and inhibit precursor reactions leading to its formation (Kim et al. 2017). Liao reported a maximum NDMA degradation rate of 17.76% for LAB isolated from traditional fermented fish products, whereas the strain *L. plantarum* AL6‐1 screened in this study exhibited a significantly higher NDMA degradation efficiency of 73.6% (Liao et al. 2019). Nguyen achieved survival rates of 28.7% and 14.0% (Nguyen et al. 2020) in simulated intestinal and gastric fluids, respectively, by encapsulating *L. plantarum* , whereas our strain, *L. plantarum* AL6‐1, demonstrated superior survival rates of 58.21% and 61.32% under comparable conditions. Additionally, *L. plantarum* AL6‐1 adhered effectively to Caco‐2 cells with an adhesion rate of 10.18% ± 0.23%, comparable to the LAB isolated from Harbin dry sausage reported by Han et al. (2017). Moreover, *L. plantarum* AL6‐1 was sensitive to clindamycin, chloramphenicol, erythromycin, tetracycline, and ampicillin, aligning closely with findings by El Issaoui et al. (2021).

Accordingly, we undertook whole‐genome sequencing and gene annotation of *L. plantarum* AL6‐1, identifying several acid‐ and bile‐tolerance determinants—GroES, GroEL, GpE, Hsp33, DnaK/DnaJ, and FtsH—that may underpin the strain's survival and colonization stability in the intestinal milieu. Comparable loci have likewise been documented in several *L. plantarum* strains—most notably PMO 08 (Oh et al. 2022) and ILSF15 (Arjun et al. 2024)—and are consistent with the in vitro acid tolerance (pH 2.0 to 4.0) and 0.3% bile‐salt tolerance exhibited by those strains. In addition, whole‐genome analysis of AL6‐1 revealed seven adhesion‐related FOF1‐ATPase proteins, five mucin‐binding domain proteins, and two proteins harboring collagen‐binding domains, further substantiating its adhesive capacity in agreement with our in vitro results and the observations of Boucard et al. (2022). Moreover, antibiotic‐susceptibility testing showed that the strain remained sensitive to several clinically important antibiotics, including amoxicillin, erythromycin, and tetracycline, indicating an overall acceptable resistance risk. Beyond the probiotic‐related loci described above, whole‐genome annotation of *L. plantarum* AL6‐1 disclosed multiple metabolic pathways intimately linked to hepatoprotection—namely NDMA catabolism, antioxidant defense, energy metabolism, and cellular repair. Of particular note, chromosomal genes encoding ALDH, which detoxifies the formaldehyde generated during NDMA degradation, were identified alongside a suite of antioxidant and DNA‐repair genes. Formaldehyde produced from NDMA is converted by ALDH into CO_2_ and H_2_, thereby attenuating the toxicity of this intermediate. In addition, genes for glutathione peroxidase, catalase, and thioredoxin–disulfide reductase provide reactive‐oxygen‐species scavenging capacity, helping to maintain intracellular redox homeostasis and consequently mitigate NDMA‐induced oxidative‐stress damage (Sasoni et al. 2016; Ali et al. 2020). Similar ALDH dominant detoxification and antioxidant pathways were confirmed in the alcoholic liver injury model of *L. plantarum* HFY09, which can significantly upregulate the expressions of SOD, GSH PX, and cat, alleviate oxidative stress, and improve liver function (Gan et al. 2021). The synergism of these genes provides molecular evidence for the function of *L. plantarum* AL6‐1 in liver protection.

To clarify the potential effect of *L. plantarum* AL6‐1 on NDMA‐induced liver injury, we measured serum transaminases (ALT, AST), calculated the liver index, and examined H&E‐stained histopathology in a mouse model. The results showed that after 12 weeks of feeding, serum AST and ALT levels in the NH group were significantly reduced compared with the NO group (*p* < 0.05). H&E staining of liver tissue showed that structural damage and inflammatory responses were markedly alleviated in the NH group. Compared with the CO group, there was no significant difference in liver index in NH mice (*p* > 0.05). In contrast, all indicators in the heat‐inactivated strain group (NS) showed no significant difference from those in the NO group (*p* > 0.05), indicating that only viable *L. plantarum* AL6‐1 could alleviate NDMA‐induced liver injury. This result is consistent with the findings of Chen et al. (2022) that *L. plantarum* Lp‐2 reduced transaminase levels and improved histological damage in a lipopolysaccharide‐induced liver injury model. However, in a CCl_4_‐induced liver injury model, Chen observed that heat‐inactivated bacteria, although less protective than live bacteria, still significantly lowered ALT and AST levels and mitigated liver tissue damage. In contrast, the heat‐inactivated *L. plantarum* AL6‐1 in this study showed no protective effect, suggesting that the alleviation of NDMA‐induced liver injury by *L. plantarum* AL6‐1 is likely dependent on its active metabolism and colonization ability, rather than nonspecific effects mediated by cell structure or physical adsorption.

MDA is a major product of lipid peroxidation, and its accumulation can disrupt cell membrane structure, induce hepatocyte necrosis, and serve as an indicator of liver injury severity (Alic et al. 2022). By measuring MDA, T‐SOD, and GSH‐Px levels in mouse serum, it was found that *L. plantarum* AL6‐1 effectively reduced MDA concentrations while enhancing the activities of T‐SOD and GSH‐Px, thereby alleviating lipid peroxidation and mitigating liver injury caused by NDMA‐contaminated sausage. These findings are consistent with those reported by Ma et al., who demonstrated that *L. plantarum* CCFM8661 reduced hepatic and renal MDA levels and enhanced SOD, CAT, and T‐AOC activities in a multi‐heavy‐metal toxicity model involving Pb, Cd, Hg, Cr, and As, ultimately relieving tissue damage induced by compound oxidative stress (Ma, Lin, et al. 2024; Ma, Wu, et al. 2024). Li et al. found that *L. plantarum* J26 significantly alleviated alcohol‐induced hepatitis and oxidative stress in mice by maintaining intestinal barrier integrity and inhibiting the TLR4‐MAPK signaling pathway (Li et al. 2022). *L. plantarum* strains from different sources have been shown to protect the liver by alleviating lipid peroxidation. The antioxidant activity of *L. plantarum* AL6‐1 may be attributed to its antioxidant‐related genes, which confer the ability to scavenge free radicals and provide cellular protection against oxidative stress‐induced damage (Ali et al. 2020).

NDMA is primarily absorbed in the upper small intestine and transported to the liver through the bloodstream for further metabolism. After feeding mice NDMA‐excess diets, plasma NDMA concentrations in the NO and NS groups were significantly higher than those in the CO group (*p* < 0.05). However, mice treated with *L. plantarum* AL6‐1 exhibited significantly reduced plasma NDMA concentrations (*p* < 0.05). Shao further demonstrated that *Lactobacillus pentosus* R3 could significantly inhibit NDMA formation by directly degrading NDMA and reducing its precursor amines, such as dimethylamine and putrescine (Shao et al. 2021). Similarly, the present study found that *L. plantarum* AL6‐1 also possesses a high NDMA‐degrading capacity. Coupled with its strong tolerance and adhesion ability, the strain can exert sustained activity in the intestinal environment. Whole‐genome analysis further supports this mechanism. Whole‐genome analysis indicates that *L. plantarum* AL6‐1 encodes genes associated with NDMA degradation and DNA repair, enabling the conversion of NDMA‐derived formaldehyde into CO_2_ and H_2_ (Dalle‐Donne et al. 2006), thereby mitigating nitrite‐induced genetic damage to some extent. Additionally, the strain encodes numerous genes related to acid and bile salt tolerance and intestinal adhesion (Table S2), enhancing its adaptability and stability in the gut and further promoting the reduction of plasma NDMA concentrations.

NDMA, as a carcinogen, relies on the catalytic activation of metabolic enzymes such as CYP2E1, CYP1A2, CYP2C37, CYP3A11, and PXR. Abnormal expression or activity of these enzymes can lead to liver damage (Wang et al. 2017). In this study, compared to the CO group, the expression of CYP2E1 in the liver of mice in the NO group was significantly upregulated, while CYP1A2 expression was significantly downregulated (*p* < 0.05), indicating that NDMA metabolism induced liver damage. However, the gene expression levels of CYP2E1 and CYP1A2 in the NH group showed no significant difference compared to the CO group (*p* > 0.05). These findings are consistent with the study by Teng et al., who demonstrated that *L. plantarum* LP104, in a high‐fat diet mouse model, activated the AMPK/Nrf2 pathway and suppressed CYP2E1 expression, thereby reducing lipid peroxidation and hepatic dysfunction (Teng et al. 2020). These findings suggest that LAB can alleviate liver injury by modulating the expression of key metabolic enzymes. Additionally, the strain modulated the expression of the CYP2C37 gene, potentially reducing the production of toxic metabolites and further supporting its liver‐protective effects (Fekete et al. 2021). Although the expression of CYP3A11 and PXR was not significantly influenced by the strain, *L. plantarum* AL6‐1 played a critical role in mitigating liver damage by regulating other metabolic enzymes and inhibiting NDMA activation. These findings suggest that the strain reduces NDMA‐induced liver damage through the modulation of multiple metabolic enzyme pathways.

Although this study demonstrated the potential of *L. plantarum* AL6‐1 in alleviating NDMA‐induced hepatotoxicity, it lacks functional validation of key metabolic enzyme expression and activity, as well as dynamic tracking of NDMA metabolites. Moreover, the strain's regulatory effects on gut microbiota, mucosal barrier integrity, and host inflammation–oxidative stress pathways have not been systematically evaluated. Future research will integrate proteomics, enzymatic activity assays, and stable isotope tracing to clarify CYP enzyme function and delineate the complete NDMA metabolic pathway. In parallel, the strain's protective effects on oxidative stress, inflammation, and DNA damage repair in the context of the gut environment will be assessed. Additionally, the combined use of gut metagenomics and metabolomics will enable deeper insights into the “microbiota–host–immune–metabolic” interaction network, ultimately supporting clinical trials to evaluate its application potential.

## 5 Conclusions

This study identified multiple key genes in *L. plantarum* AL6‐1 through whole‐genome sequencing, including genes involved in NDMA metabolism, antioxidation, DNA repair, and metabolic regulation. Animal experiments demonstrated that *L. plantarum* AL6‐1 significantly reduced the liver index and serum AST and ALT levels, alleviating liver cell swelling, disrupted hepatic cord arrangement, and inflammatory cell infiltration induced by excessive NDMA intake in mice. Additionally, the strain decreased liver MDA levels while enhancing T‐SOD and GSH‐Px activities. Furthermore, *L. plantarum* AL6‐1 regulated the expression of CYP2E1, CYP1A2, and CYP2C37, inhibiting NDMA metabolic activation and reducing NDMA concentrations in mouse plasma. These findings provide crucial theoretical support for the potential role of *L. plantarum* AL6‐1 in preventing and mitigating liver damage caused by excessive NDMA intake, laying a solid foundation for its development as a probiotic.

## Author Contributions

**Erke Sun:** conceptualization (equal), data curation (equal), investigation (equal), methodology (equal), writing – original draft (equal), writing – review and editing (equal). **Xueying Sun:** data curation (equal), investigation (equal). **Jin Guo:** data curation (equal), validation (equal). **Lina Sun:** investigation (equal), supervision (equal). **Ye Jin:** methodology (equal), project administration (equal). **Lin Su:** funding acquisition (equal), writing – review and editing (equal). **Lihua Zhao:** funding acquisition (equal), writing – review and editing (equal).

## Ethics Statement

All animal experiments were conducted in compliance with the guidelines of the Animal Experiment Ethics Committee of Inner Mongolia Agricultural University (Approval No.: NND2021072), in compliance with the Declaration of Helsinki and relevant national regulations.

## Conflicts of Interest

The authors declare no conflicts of interest.

## Supporting information


**Table S1:** Decreasing effect of different strains on concentrations of NDMA.
**Table S2:** Genome‐wide information of the strain.
**Table S3:** Environmental adaptability related genes.

## Data Availability

The data that support the findings of this study are available from the corresponding author upon reasonable request.
