# Supplementary material for: A Lactiplantibacillus plantarum AL6‐1 Strain Isolated From Air‐Dried Meat Mitigates N‐Dimethylnitrosamine‐Induced Hepatic Injury in Mice
Source: Food Sci Nutr. 2025 Sep 15;13(9):e70861. doi: 10.1002/fsn3.70861 (PMC12434481; doi:10.1002/fsn3.70861)
Supplement: Supplementary file 1 — Table S1: Decreasing effect of different strains on concentrations of NDMA. Table S2: Genome‐wide information of the strain. Table S3: Environmental adaptability related genes. [file FSN3-13-e70861-s001.docx]

**Appendices：**

**Table.S1** Decreasing effect of different strains on concentrations of NDMA

| Strain Name | Strain Number | Degradation Rate (%) |
| --- | --- | --- |
| *Lactiplantibacillus plantarum* | 30X-15 | 44.15±3.86 |
|  | 37X-2 | 29.24±2.57 |
|  | 37X-6 | 47.13±3.6 |
|  | AL6-1 | 73.62±2.25 |
|  | AL6-6 | 37.71±2.33 |
|  | BL4-7 | 46.64±3.05 |
|  | BL4-8 | 26.97±2.23 |
|  | CL4-3 | 51.98±2.7 |
|  | ML2-2 | 45.17±2.12 |
|  | ML3-1 | 44.67±2.00 |
|  | X3-1 | 16.81±4.08 |
|  | X3-9 | 8.5±2.46 |
|  | X3-10 | 10.86±3.32 |
|  | X3-12 | 15.84±2.85 |
|  | X3-2B | 5.55±1.35 |
|  | X3-3B | 41.88±3.15 |
|  | X3-5B | 21.14±3.26 |
|  | PL-4 | 37.8±3.00 |
| *Pediococcus pentosaceus* | 30X-1 | 43.17±3.77 |
|  | 30X-3 | 40.64±2.68 |
|  | 30X-4 | 54.2±2.84 |
|  | 30X-5 | 41.36±1.92 |
|  | 30X-6 | 36.96±2.36 |
|  | 30X-7 | 32.82±3.15 |
|  | 30X-9 | 37.1±1.83 |
|  | 30X-10 | 35.9±1.96 |
|  | 30X-11 | 56.76±3.07 |
|  | 30X-13 | 47.77±2.74 |
|  | 37X-1 | 25.12±1.62 |
|  | 37X-3 | 40.88±3.77 |
|  | 37X-4 | 46.3±3.43 |
|  | 37X-5 | 48.08±4.84 |
|  | 37X-7 | 43.39±1.47 |
|  | 37X-8 | 45.67±2.28 |
|  | 37X-9 | 44.8±4.13 |
|  | 37X-10 | 37.59±2.61 |
|  | 37X-11 | 68.93±4.02 |
|  | 37X-12 | 46.76±3.71 |
|  | 37X-13 | 61.95±5.09 |
|  | 37X-14 | 52.62±4.05 |
|  | 37X-15 | 37.86±2.61 |
|  | QL-1 | 6.57±2.95 |
|  | QL-3 | 52.73±3.4 |
|  | RL-1 | 23.4±2.57 |
|  | TL-3 | 50.86±5.92 |
|  | X3-13B | 24.88±2.2 |
| *Lactobacillus helveticus* | CL4-1 | 32.6±1.75 |
|  | SL-1 | 66.51±5.96 |
|  | SL-2 | 54.89±2.28 |
|  | SL-3 | 52.97±2.11 |
| *Latilactobacillus curvatus* | HX2-4 | 31.69±2.7 |
|  | X3-1B | 8.01±2.29 |
| *Pediococcus acidilactici* | X3-4B | 32.05±2.86 |

**Table.S2** Genome-wide information of the strain

| Properties | Value | GC content（%） |
| --- | --- | --- |
| Genome size (bp) | 3295614 | 44.41 |
| Chromosome(bp) | 3017413 | 44.80 |
| PlasmidA (bp) | 70756 | 41.35 |
| PlasmidB (bp) | 58663 | 39.46 |
| PlasmidC (bp) | 57578 | 39.80 |
| PlasmidD (bp) | 46272 | 41.21 |
| PlasmidE (bp) | 44932 | 38.59 |
| tRNA | 68 | - |
| rRNA | 16 | - |
| sRNA | 48 | - |

**Table.S3** Environmental adaptability related genes

| Location | Gene names | Gene ID | Reference gene sequences |
| --- | --- | --- | --- |
| Chromosome | GroES | gene0560 | CP138486.1 |
|  | GroEL | gene0561 | CP143285.1 |
|  | GrpE | gene1556 | CP143285.1 |
|  | Hsp33 | gene0446 | CP032648.1 |
|  | DnaK | gene1555 | CP166731.1 |
|  | DnaJ | gene1554 | CP032648.1 |
|  | FtsH | gene0445 | CP103912.1 |
|  | Pta | gene1746 | CP021997.1 |
|  |  | gene1747 | CP028334.1 |
|  |  | gene1902 | CP035156.1 |
|  | AckA | gene0181 | CP150141.1 |
|  |  | gene0273 | CP110799.1 |
|  |  | gene1831 | CP016071.1 |
|  | F0F1-ATPase | gene1936 | CP143285.1 |
|  |  | gene1937 | CP084715.1 |
|  |  | gene1938 | CP103912.1 |
|  |  | gene1939 | CP035156.1 |
|  |  | gene1941 | CP103912.1 |
|  |  | gene1942 | CP138486.1 |
|  |  | gene1943 | CP143285.1 |
|  | MucBP | Gene0329 | CP150141.1 |
|  |  | Gene0747 | CP143285.1 |
|  |  | Gene0964 | CP096855.1 |
|  |  | gene1992 | CP139818.1 |
|  |  | Gene2498 | CP020093.1 |
|  | Collagen binding domains | gene1606 | CP060172.1 |
|  |  | gene2397 | CP084715.1 |
